# The Impact of Gunshot Wounds on Healthcare Utilization in Trauma Patients: Retrospective Review of Trauma Centers in the United States

**DOI:** 10.7759/cureus.103857

**Published:** 2026-02-18

**Authors:** Theodore Lainiotis, Ria Sebastian, Mark Mckenney, Ilko Luque

**Affiliations:** 1 Osteopathic Medicine, Nova Southeastern University Dr. Kiran C. Patel College of Osteopathic Medicine, Davie, USA; 2 Surgery, University of South Florida, Morsani School of Medicine, Tampa, USA; 3 Trauma and Acute Care Surgery, Hospital Corporation of America (HCA) Florida Kendall Hospital, Miami, USA

**Keywords:** gunshot wounds, health care resources utilization, national trauma data bank, public health, trauma

## Abstract

Background

Gunshot wounds (GSW) contribute to poor patient outcomes and increased consumption of healthcare resources. The literature has expanded on the costs of treating patients with firearm injuries. However, further research is needed to understand the implications of GSW on healthcare resource utilization in trauma care. This study aims to compare hospital and post-hospital resource utilization between GSW and non-GSW trauma patients in the United States (US) trauma centers.

Methods

The National Trauma Data Bank (NTDB) was used to collect data retrospectively (2019-2021) on adult patients (age ≥18) admitted to US level 1 or 2 trauma centers. Bivariate and multivariable analyses were conducted to assess whether mode of transportation, health insurance, length of stay, or post-hospital discharge disposition were significantly different in GSW patients compared to non-GSW patients. Statistical significance was defined as p<0.05.

Results

We identified 1,596,079 trauma patients, of whom 106,844 were GSW patients (6.7%), and 1,489,235 were non-GSW patients (93.3%). GSW patients were transported by helicopter to a hospital more often compared to non-GSW patients for care (10.9% vs 8.1%) (X^2^ (1) = 928.6, p=5.9^-204^). Uninsured GSW patients ended up at a hospital with more frequency compared to uninsured non-GSW patients (31.9% vs 9.9%) (X^2^ (1) = 45,622.9, p<0.001). Healthcare was covered with government-funded insurance in GSW patients more often compared to non-GSW patients (66.9% vs 62.4%) (X^2 ^(1) = 586.4, p = 1.5^-129^). A significant difference in the intensive care unit's length of stay was observed between the two groups (*U* = 7,260,338,161.0, *Z* = 7.6, p = 2.5^-14^). The mean rank for the GSW group was 220,491.8, whereas the mean rank for the non-GSW group was 215,328.7. The multivariable analysis indicated that GSW patients had increased odds of discharge to a psychiatric hospital or psychiatric distinct part unit of a hospital (aOR 1.5, CI 1.4 - 1.6, p=4.814^-41^) compared to non-GSW patients, when adjusting for age, sex, and mental/personality disorders' status.

Conclusion

GSW trauma is associated with high demands on acute care resources and significant gaps in post-hospital discharge support. GSW trauma underscores the importance of equitable, data-driven resource allocation strategies. A comprehensive and equity-oriented approach is warranted to enhance the efficiency and responsiveness of trauma care systems.

## Introduction

Gunshot wounds (GSW) remain a major public health crisis in the United States (US), contributing significantly to morbidity, mortality, and the strain placed on healthcare resources. Each year, over 40,000 firearm-related deaths and approximately 80,000 nonfatal GSWs are reported nationwide [1]. Survivors often require intensive and prolonged medical intervention, including emergency transport, acute surgical care, hospital admissions, intensive care unit (ICU) length of stay (LOS), and possibly ongoing rehabilitation services. While the financial implications of firearm injuries have been previously documented, less attention has been given to the patterns of healthcare resource utilization and the systemic demands placed on trauma care infrastructure across the entire continuum of care [2].

The burden of GSW trauma is characterized by high resource utilization, including advanced life support and air medical transport, and continues through extended hospitalizations and critical care admissions. ICU admissions, which are among the most resource-intensive components of trauma care, are common among severe GSW patients. Beyond the acute setting, survivors often require complex post-hospital discharge planning. Patients are frequently transitioned to post-acute care settings such as a skilled nursing facility (SNF), a long-term acute care hospital (LTACH), or psychiatric services due to physical, neurological, or psychological sequelae [3,4]. These facilities must accommodate patients with high levels of dependency and specialized care needs, contributing to broader care coordination issues and prolonged resource utilization with a significant financial burden to the community.

Despite the intensity of care required, the allocation and availability of appropriate post-hospital discharge resources for GSW survivors remain inconsistent. Access to rehabilitation and long-term care is often influenced by factors such as insurance status or mental health comorbidities, leading to potential disparities in outcomes and increased risk for readmission [3,5]. Understanding how GSW trauma patients utilize hospital and post-acute resources is critical for designing systems that equitably and efficiently meet patient needs.

This study aims to compare hospital and post-hospital resource utilization between GSW and non-GSW trauma patients. We hypothesize that GSW patients will have higher utilization of hospital and post-hospital resources compared to non-GSW patients.

## Materials and methods

This study received institutional review board exemption because our retrospective research involved analysis of de-identified data. Patients aged 18 years or older and admitted to level 1 or 2 trauma centers were included. Patients younger than 18 years old or admitted to level 3 trauma centers were excluded.

The National Trauma Data Bank (NTDB) was used to collect data retrospectively (2019-2021) on trauma patients. The NTDB is a repository of trauma mechanisms and outcomes from U.S. trauma centers, which includes demographic information, mechanism of injury, pre-existing conditions, and outcomes. The NTDB is a representation of "real world" data for trauma patients admitted for care in the United States trauma centers. All trauma hospitals accredited by the American College of Surgeons Committee on Trauma (ACSCOT) follow the National Trauma Data Standard (NTDS) to collect data on trauma patients, regardless of the level of accreditation/designation. The standard data dictionary minimizes variation and misclassification of data between hospital-based registries. The NTDB has been used as a valid source for research to improve the care of injured patients. 

The variables collected were age, sex, primary method of payment, pre-existing mental/personality disorders' status, mechanism of injury, injury severity score (ISS), mode of transportation, and trauma center's level. The outcomes evaluated were post-hospital discharge disposition, hospital (H), and ICU-LOS.

The NTDB defines "firearm" as a cause of injury event in the "mechanism" variable. We created a new variable "GSW status"; patients with firearm injury were grouped in the "GSW" category, and patients with any mechanism of injury other than firearm were grouped in the "non-GSW" category.

The NTDB specifies pre-existing mental/personality disorders as the presence of pre-injury depressive disorder, bipolar disorder, schizophrenia, borderline or antisocial personality disorder, and/or adjustment disorder/post-traumatic stress disorder.

The primary method of payment is the source of payment patients use for hospital care. The NTDB defines the primary method of payment as Medicaid, Medicare, other government-funded insurance, self-pay, or private/commercial insurance. We created a new variable "health insurance status"; patients with Medicaid, Medicare, other government-funded insurance, or private/commercial insurance were grouped in the "insured" category, and patients whose primary method of payment was self-pay were grouped in the "uninsured" category. Then, another variable, "health insurance type", was created; patients with Medicaid, Medicare, or other government-funded insurance were grouped in the "government-funded" health insurance category, and patients with private/commercial insurance were grouped in the "private" health insurance category. Patients with the self-pay method were declared "missing" (using user-defined missing value 9999) when the variable "health insurance type" was created. 

Bivariate analysis

We compared GSW patients and non-GSW patients to assess whether the difference in the mode utilized (helicopter vs ambulance) to transport those patients to a trauma center was statistically significant. Another analysis was conducted to assess the association between GSW status (GSW/non-GSW) and health insurance status (insured/uninsured). Next, we evaluated whether the type of health insurance (government-funded/private) in GSW patients was statistically different compared to non-GSW patients. The Chi-squared test or Fisher's exact test was used to analyze the data for statistical significance. The Chi-squared test was used for those categorical variables where at most 20% of the expected frequencies were less than five; otherwise, Fisher's exact test was used. 

The Mann-Whitney test was used to evaluate the association between GSW status (GSW/non-GSW) and H-LOS and ICU-LOS. The non-parametric statistical test compared H-LOS or ICU-LOS between two independent groups. In addition, the distribution of the values for H-LOS or ICU-LOS was not normal for the GSW and non-GSW cohorts.

Multivariable analysis

Multiple logistic regression was used to assess the association between GSW status (GSW/non-GSW) and discharge to inpatient rehabilitation or designated unit, SNF, LTACH, or psychiatric hospital or psychiatric distinct part unit of a hospital, when adjusting for age, sex, pre-existing mental/personality disorders' status, and ISS (continuous). Sex, increased age, or ISS may be associated with higher odds of discharge to inpatient rehabilitation or designated unit, SNF, or LTACH in GSW patients. In addition, the history of mental/personality disorders in these patients may influence discharge for mental health care.

Patients' ISS was categorized as mild (1-8), moderate (9-15), severe (16-24), or critically injured (≥25). The categorization of injury severity has been employed to examine clinical outcomes in trauma research. A retrospective multicenter study (1998-2003) in the US showed that trauma patients with mild (0-8), moderate (9-15), or serious (≥16) injuries had increased specialized resource use [6].

Statistical significance was defined as a p-value less than 0.05 with a 95% confidence interval (CI). The statistical software used for analysis was SPSS Version 29.0 (IBM Inc., Armonk, New York). 

Management of missing data 

User-defined missing value (9999) was the method to manage missing data. Data was declared as "missing" for the subsequent variables based on these criteria: sex - no information was available, or patients identified as non-binary; primary method of payment - patients who were not billed (for any reason), or had other method of payment that was not specified; ISS, transportation's mode, or post-hospital discharge disposition - no information was available. Pre-existing mental/personality disorders' status - information was not known/not recorded.

## Results

This study included a total of 1,596,079 adult trauma patients who were admitted to a level 1 or 2 trauma center between 2019 and 2021, of whom 106,844 were GSW patients (6.7%) and 1,489,235 were non-GSW patients (93.3%).

Most cases of GSW occurred in males (86.5%). GSW patients were significantly younger (median 29, IQR 18 - 89) than non-GSW patients (median 60, IQR 1 - 89) (U = 28,994,538,350.5, Z = -347.6, p<0.001). GSW patients were transported by helicopter to a hospital more often compared to non-GSW patients for care (10.9% vs 8.1%) (X2 (1) = 928.6, p=5.9-204). GSW patients were frequently transported to a level 1 trauma center (74.0%) (Table 1). 

**Table 1:** Characteristics of patients *Statistical significance (p < 0.05) is indicated with this variable for GSW patients compared to non-GSW patients. **Data declared as "missing" is reported for the following variables: sex - 622 patients in the GSW group and 5417 patients in the non-GSW group; health insurance's status - 5469 patients in the GSW group and 59,851 patients in the non-GSW group; health insurance’s type – 37,785 patients in the GSW group and 200,975 in the non-GSW group; pre-existing mental/personality disorders' status - 102,151 patients in the GSW group and 26,143 in the non-GSW group; ISS - 86 patients in the GSW group and 1783 in the non-GSW group; and helicopter’s transportation - 235 in the in the GSW group and 3713 in the non-GSW group; and transportation's mode - 15,916 in the GSW group and 187,468 in the non-GSW group. GSW - gunshot wounds; ISS - injury severity score

Variables	GSW group (n = 106,844)	Non-GSW group (n = 1,489,235)	p-value
Demographics			
Age, median (IQR)	29 (1 - 89)	60 (1 - 89)	<0.001^*^
Sex^**^, n (%)			
Female	13,780 (13.0)	637,145 (42.9)	<0.001^*^
Male	92,442 (87.0)	846,673 (57.1)	
Health insurance status^**^, n (%)			
Uninsured	32,316 (31.9)	141,124 (9.9)	<0.001^*^
Insured	69,059 (68.1)	1,288,260 (90.1)	
Health insurance type^**^, n (%)			
Government	46,225 (66.9)	803,342 (62.4)	1.528^-129*^
Private	22,834 (33.1)	484,918 (37.6)	
Pre-existing condition, n (%)			
Mental/personality disorder status^**^			
No	93,602 (91.6)	1,267,559 (86.6)	<0.001^*^
Yes	8,549 (8.4)	195,533 (13.4)	
Injury profile, n (%)			
Injury severity^**^			
Mild (ISS: 1-8)			
No	64,893 (60.8)	821,347 (55.2)	7.016^-274*^
Yes	41,865 (39.2)	666,105 (44.8)	
Moderate (ISS: 9-15)			
No	74,029 (69.3)	931,294 (62.6)	<0.001^*^
Yes	32,729 (30.7)	556,158 (37.4)	
Severe (ISS: 16-24)			
No	93,502 (87.6)	1,328,244 (89.3)	6.878^-68*^
Yes	13,256 (12.4)	159,208 (10.7)	
Critically (ISS: ≥25)			
No	87,850 (82.3)	1,381,471 (92.9)	<0.001^*^
Yes	18,908 (17.7)	105,981 (7.1)	
Transportation mode^**^			
Helicopter	9939 (10.9)	104,868 (8.1)	5.860^-204*^
Ambulance	80,989 (89.1)	1,196,899 (91.9)	
Trauma center level			
I	79,023 (74.0)	902,726 (60.6)	<0.001^*^
II	27,821 (26.0)	586,509 (39.4)	

Uninsured GSW patients ended up at a hospital with more frequency compared to uninsured non-GSW patients (31.9% vs 9.9%) (X2 (1) = 45,622.9, p<0.001). Healthcare was covered with government-funded health insurance in GSW patients more often compared to non-GSW patients (66.9% vs 62.4%) (X2 (1) = 586.4, p=1.5-129) (Table 1). Table 2 shows the count and percentage for post-hospital discharge disposition for the GSW patients and non-GSW patients.

**Table 2:** Count and percentage for post-hospital discharge disposition *Statistical significance (p<0.05) is indicated with this variable for GSW patients compared to non-GSW patients. **Data declared as "missing" for post-hospital discharge disposition is reported for 82,672 patients in the GSW group and 157,536 patients in the non-GSW group. GSW - gunshot wounds

Variables	GSW group (n = 106,844)	Non-GSW group (n = 1,489,235)	p-value
Post-hospital discharge disposition^** ^n (%)			
Inpatient rehabilitation or designated unit			
No	78,050 (94.4)	1,161,369 (87.2)	< 0.001^*^
Yes	4622 (5.6)	170,330 (12.8)	
Skilled nursing facility			
No	81,361 (98.4)	1,116,896 (83.9)	< 0.001^*^
Yes	1311 (1.6)	214,803 (16.1)	
Long-term care acute hospital			
No	81,998 (99.2)	1,320,794 (99.2)	0.911
Yes	674 (0.8)	10,905 (0.8)	
Psychiatric hospital or psychiatric distinct part unit of a hospital			
No	81,163 (98.2)	1,321,742 (99.3)	1.927^-246*^
Yes	1509 (1.8)	9957 (0.7)	

Significant difference in H-LOS was observed between the two groups (U = 67,834,869,882.0, Z = -68.7, p<0.001). The mean rank for the GSW group was 698,450.8, while the mean rank for the non-GSW group was 798,049.9. These findings suggest GSW patients had a shorter H-LOS compared to non-GSW patients. ICU-LOS was also significantly different between the two groups (U = 7,260,338,161.0, Z = 7.6, p=2.5-14). The mean rank for the GSW group was 220,491.8, whereas the mean rank for the non-GSW group was 215,328.7. These findings indicate that GSW patients had a longer ICU-LOS compared to non-GSW patients.

Table 3 shows the adjusted odds ratio (aOR) and confidence interval (CI) for the association between GSW status (GSW/non-GSW) and post-hospital discharge disposition. We showed that GSW patients had increased odds of discharge to a psychiatric hospital or psychiatric distinct part unit of a hospital (aOR 1.50, CI 1.41 - 1.59, p=4.814-41) compared to non-GSW patients, when adjusting for age, sex, and mental/personality disorders' status. The odds of referral for mental health care increased by 50% in GSW patients. However, GSW patients had decreased odds of discharge to an inpatient rehabilitation or designated unit (aOR 0.59, CI 0.57 - 0.61, p=5.807-228), SNF (aOR 0.46, CI 0.44 - 0.49, p=1.238-155), or LTACH (aOR 0.77, CI 0.71 - 0.84, p=2.659-9) compared to non-GSW patients, when adjusting for age, sex, and ISS.

**Table 3:** Multivariable analysis to examine the association between GSW status (GSW/non-GSW) and post-hospital discharge disposition *Multiple logistic regression was used to calculate aOR for post-hospital discharge disposition, when adjusting for age, ISS, sex, or pre-existing mental/personality disorder status. GSW - gunshot wounds; ISS - injury severity score; aOR - adjusted odds ratio; CI - confidence interval

Variables	Inpatient rehabilitation or designated unit			Skilled nursing facility			Long-term care acute hospital			Psychiatric hospital or psychiatric distinct part unit of a hospital		
	aOR^*^	95% CI	p-value^*^	aOR	95% CI	p-value	aOR	95% CI	p-value	aOR	95% CI	p-value
GSW status (main variable)	0.59	0.574, 0.612	5.807^-228 ^	0.46	0.439, 0.491	1.238^-155^	0.77	0.708, 0.840	2.659^-9^	1.5	1.413, 1.591	4.814^-41^
Control variables												
Age	1.02	1.020, 1.020	<0.001	1.06	1.061, 1.061	<0.001	1.01	1.011, 1.013	1.295^-118^	0.96	0.962, 0.964	<0.001
ISS (continuous)	1.05	1.048, 1.049	<0.001	1.01	1.004, 1.005	1.179^-38^	1.08	1.079, 1.082	<0.001	-	-	-
Male (Female: reference)	0.85	0.844, 0.862	5.631^-188^	0.68	0.673, 0.687	<0.001	1.3	1.247, 1.353	4.226^-36^	1.61	1.543, 1.679	6.784^-109^
Mental/personality disorders’ status	-	-	-	-	-	-	-	-	-	18.82	18.057, 19.615	<0.001

## Discussion

This NTDB analysis offers a broad evaluation of healthcare resource utilization among GSW patients in the US. Consistent with prior work, GSW patients demonstrated substantially greater acute care needs than non-GSW patients, particularly reflected in prolonged ICU-LOS. Given that ICU capacity represents a finite, high-acuity resource, extended utilization by GSW patients highlights the disproportionate demand for firearm injuries placed on trauma systems and may exacerbate the ongoing challenges in bed availability. Prolonged hospitalization further reflects the intensive clinical demands of penetrating trauma, including increased physician oversight and extended bedside nursing care. This aligns with the greater injury severity, operative complexity, and management challenges inherent to firearm-related trauma described in literature [2,4]. Given the cross-sectional and retrospective nature of the NTDB, these findings reflect associations rather than causal relationships. Interpretations regarding resource strain and downstream care access should therefore be viewed as contextualized implications rather than direct effects. 

The higher utilization of helicopter transport in GSW patients compared to non-GSW (10.9% vs 8.1%) further illustrates the urgent nature of firearm injuries and the subsequent reliance on advanced pre-hospital interventions. Air medical transport represents a considerable deployment of limited trauma system resources, requiring coordination, trained personnel, and rapid access to high-level trauma centers. These findings indicate the burden on both pre-hospital and inpatient resources to meet the needs of firearm trauma, often diverting services from other emergent or elective procedures [1]. Prior analyses have highlighted that patients with firearm injuries experience longer operative times and higher rates of unplanned reoperations, further amplifying acute care utilization [7]. Our analysis also found that GSW patients were more likely to be uninsured or reliant on public insurance, reinforcing the intersection of these injuries with resource-limited populations. 

Interestingly, despite their elevated need for acute care, GSW patients were less likely to be discharged to inpatient rehabilitation, SNF, or LTACH than non-GSW patients. While this may partly be attributed to injury characteristics, it also reflects possible challenges in downstream resource availability and access. GSW patients are disproportionately associated with high-intensity services, yet may face barriers when transitioning to post-acute settings, raising concerns about potential inequities in trauma system recovery pathways [3,5]. Misalignment between clinical need and post-hospital discharge disposition contributes to inefficient healthcare utilization and may increase the risk of complications, readmissions, and prolonged recovery [1,2]. Insurance status is a well-documented determinant of healthcare access, and gaps in coverage can result in suboptimal placements or delayed transitions of care. These findings are consistent with longstanding evidence linking firearm injury, socioeconomic disadvantages, and reduced access to post-acute services [8]. Post-hospital discharge disposition after firearm injury reflects a spectrum of recovery needs, spanning both physical and psychological domains.

The significantly higher odds of discharge to psychiatric care in GSW patients, even when adjusting for pre-existing mental/personality disorders' status, further highlight the psychological burden of firearm trauma previously described. While the NTDB does not permit differentiation between psychiatric admissions related to injury intent versus post-traumatic psychological sequelae, the observed association suggests a heightened mental health care need among survivors. Current fragmentation between acute trauma management and mental health follow-up leaves many survivors inadequately supported, contributing to persistent psychological morbidity [5,9,10]. Integrating screening protocols, dedicated social work, behavioral health consultation, and outpatient mental health follow-up into trauma care pathways aligns with growing calls to conceptualize firearm injury as both a surgical emergency and a public health crisis [11,12]. 

Collectively, these findings highlight the resource demands associated with firearm injuries, spanning pre-hospital transport, inpatient critical care, and post-hospital discharge support. The observed differences in discharge patterns suggest that post-acute care utilization may be influenced not only by clinical need but also by non-clinical factors, including insurance status and resource availability, that limit access to long-term rehabilitation and robust mental health services [4,5]. From a public health and systems-level perspective, these results emphasize the need for an integrated approach that extends beyond surgical management and into preventive action. Efforts to mitigate firearm-related harm must include evidence-based firearm safety initiatives, violence prevention strategies, and policies that address both interpersonal and self-inflicted injury. Firearm trauma represents a convergence of acute injury, psychological distress, and social vulnerability, underscoring the need for well-staffed and funded social work departments, equitable access to rehabilitation, and increased availability to structured behavioral health follow-up. Strengthening care coordination infrastructure in these ways, particularly in trauma centers serving high-violence regions, may help ensure timely referral to appropriate post-acute and mental health services regardless of insurance status. Future research should examine how resource allocation varies across injury patterns, insurance types, and geographic regions. Together, these insights can inform efforts to build more effective, equitable, and responsive trauma systems [12]. 

This study has several limitations. As with any retrospective analysis, the results depend on the accuracy and completeness of the reported data, and missing or miscoded information may introduce bias. The dataset does not capture longitudinal outcomes, such as functional recovery, readmissions, or psychosocial reintegration, limiting conclusions beyond the indexed hospitalization. Additionally, important confounders, such as hospital-level resources, regional variation in trauma systems, and detailed socioeconomic context, could not be fully accounted for. Prospective and longitudinal studies are needed to elucidate these relationships and validate the observed associations.

## Conclusions

Firearm-related trauma is associated with increased utilization of acute care resources and distinct hospital discharge patterns that may reflect gaps in access to post-acute and rehabilitative support. In the context of high national trauma volume and limited critical care capacity, even incremental increases in length of stay can result in meaningful system-level strain and reduced bed availability. These findings underscore the need for coordinated, data-driven approaches to trauma system planning, including investment in critical care capacity, standardized discharge pathways, and increased access to rehabilitation and behavioral health services. At the policy level, these results also reinforce the importance of evidence-informed firearm injury prevention policies that address both violent and self-inflicted harm and aim to reduce the incidence and downstream healthcare burden of firearm trauma. Strengthening integration of mental health resources and addressing socioeconomic barriers to post-acute care may improve recovery and reduce morbidity among firearm injury survivors. While these findings demonstrate associations between firearm injury and healthcare resource utilization, causal inference is limited by the retrospective, cross-sectional design of the NTDB. Future research should focus on longitudinal outcomes, including recovery trajectories, community reintegration, and targeted interventions. Ultimately, a comprehensive, equity-focused trauma systems approach is essential to ensure that care for firearm injury extends beyond acute survival to support durable recovery and system sustainability. 
